# Utility of Post-Splinting Conventional Radiographs in Adult Patients With Ankle Fractures Presenting to the Emergency Department

**DOI:** 10.1177/19386400221118898

**Published:** 2022-08-25

**Authors:** Lucia Francisca Joseph Walraven, Milan Lennaert Ridderikhof, Tim Schepers

**Affiliations:** Department of Emergency Medicine, Amsterdam UMC, Location Academic Medical Center, Amsterdam, The Netherlands; Department of Emergency Medicine, Amsterdam UMC, Location Academic Medical Center, Amsterdam, The Netherlands; Department of Trauma Surgery, Amsterdam UMC, Location Academic Medical Center, Amsterdam, The Netherlands

**Keywords:** conventional radiograph and splinting, trauma fractures, ankle injuries other, emergency department

## Abstract

**Background:**

Post-splinting radiographs are often performed in patients with ankle fractures to identify displacement that potentially occurs during splinting. The objective of this study was to investigate the significance of post-splinting conventional radiographs, with an emphasis on stable ankle fractures, not requiring reduction.

**Methods:**

A retrospective study in which all adult patients presenting with ankle fractures to the emergency department of a level 1 trauma center were included. The primary outcome was frequency of displacement at post-splinting radiographs. Secondary outcome was the rate of successful reduction attempts.

**Results:**

A total of 225 patients were included and the majority had a Supination-External Rotation (SER) type 2 or Weber B ankle fracture. One hundred fifty patients (mainly SER 2 fractures [68%] or Weber B [89%] fractures), were treated with a splint without fracture reduction. Post-splinting radiographs in these patients, as well as in all patients with a Supination-Adduction (SA) type 1 and 2 fractures, did not show loss of alignment.

**Conclusion:**

Post-splinting radiographs are probably not necessary in any SA and SER type 2 or Weber A/B ankle fractures without medical clear space widening or need for reduction as no loss of alignment occurred when applying a splint.

**Level of Evidence:**

IV—Case Series

## Introduction

### Background

Ankle fractures are one of the most common fractures with which patients present to the emergency department for an estimated incidence of 187 per 100 000 patients annually.^1^ Between 40% and 75% of these fractures are stable fractures.^2-4^ By definition, stable ankle fractures are fractures that are not displaced. In addition, several authors concluded that stable fractures do not have medial clear space (MCS) widening in the conventional radiograph of the ankle joint.^5-11^

“This study suggests that post-splinting conventional radiographs for stable ankle fractures without widened MCS are unnecessary in the emergency department.”

Several classification systems have been developed previously of which the Lauge-Hansen and Weber Classification are used most commonly in daily clinical practice (see Table 1).^12-14^ These classification systems are based on nonstress radiographs and are useful for describing fracture patterns, with the Weber system focusing on anatomical position of the fracture relative to the syndesmosis and the Lauge-Hansen focusing on trauma mechanism including the injury of the medial complex and therefore for this study probably most useful.

**Table 1.** Lauge-Hansen and Weber Classification.

Weber A Lauge-Hansen *supination-adduction*	Infrasyndesmotic Type 1: Tension on the lateral collateral ligaments results in rupture of the ligaments (talofibular) or avulsion of the lateral malleolus below the syndesmosis (true Weber A) Type 2: Oblique fracture of the medial malleolus
Weber B Lauge-Hansen *supination-exorotation*	Transsyndesmotic Type 1: Rupture of anterior syndesmosis (anterior tibiofibular ligament) Type 2: Oblique fracture of fibula (true Weber B) Type 3: Rupture of posterior syndesmosis (posterior tibiofibular ligament) or fracture of posterior malleolus Type 4: Avulsion of medial malleolus or rupture of medial collateral bands (deltoid ligament)
Weber C Lauge-Hansen *pronation exorotation*	Suprasyndesmotic Type 1: Avulsion of medial malleolus or ligamentous rupture (deltoid) Type 2: Rupture of anterior syndesmosis (anterior tibiofibular ligament) Type 3: Fibula fracture above the level of syndesmosis Type 4: Avulsion of posterior malleolus or rupture of posterior syndesmoses (posterior tibiofibular ligament)

Regarding fracture treatment, a fairly comprehensive consensus opinion is that stable ankle fractures should be treated conservatively with immobilization and unstable fractures are expected to have better outcomes with surgical treatment.^8,10,15,16^

Several treatment modalities exist that can be used for immobilization, such as splinting or casting, and there is no firm evidence regarding the optimal method of immobilization.^17,18^ In daily clinical practice, a cast can be used in case reduction of the ankle fracture is necessary and a splint could be applied in stable fractures.

### Importance

In the emergency department, it is common practice to reduce displaced ankle fractures before immobilization and conventional radiographs are obtained after immobilization to ensure adequate alignment at disposition and to direct definitive treatment. However, the exact role of radiography after applying the splint in ankle fractures not requiring fracture reduction remains controversial. Theoretically, applying a splint could displace an ankle fracture or induce inadequate alignment of the ankle mortise due to manipulation while applying. Accordingly, some clinicians at least in Dutch trauma centers and perhaps in other countries as well, advocate performing radiographs of the ankle joint in all patients after applying a splint. One of the key reasons to obtain these radiographs in teaching institutions is to evaluate the position of the ankle, either after reduction, to assess the quality of the reduction, or after initially deemed stable fractures, to assure a maintaining of the correct position after manipulation of applying the cast. Others have stated that post-splinting conventional radiographs are unnecessary in nondisplaced fractures, but firm evidence is lacking.^19,20^ Authors of previous studies have focused on the use of post-splinting conventional radiographs that were performed during (outpatient) trauma clinic visits and not in the emergency department or if the study population was small.^2,19-23^

Consequently, it would be of importance to evaluate the use of post-splinting conventional radiographs in ankle fractures. Reducing the number of radiographs potentially shortens the length of stay in the emergency department, minimizes radiation exposure, and would decrease health care costs.

### Goals of The Current Study

Consequently, the aim of this study was to investigate the significance of post-splinting conventional radiographs. More specifically, the objectives were to identify how frequently secondary fracture displacement occurred after applying a splint in patients presenting to the emergency department with an ankle fracture and what the specific fracture characteristics were. The emphasis of the analysis was to first assess stable ankle fractures, not requiring reduction, and, second, how successful reduction attempts were in the emergency department.

## Methods

### Study Design and Selection of Participants

This was a retrospective study, including all adult patients, aged 18 years and older, who presented to the emergency department of a Dutch level 1 trauma center with an ankle fracture between January 1, 2012, and December 31, 2015. Strengthening the Reporting of Observational studies in Epidemiology (STROBE) guidelines were used to aid in ensuring high-quality presentation of our study.

All patients were initially treated with a below-knee, non-weight-bearing plaster splint, with sufficient padding and between 12 and 15 layers of plaster of paris at the emergency department, as was standard practice during the study period. Patients received follow-up at the outpatient clinic, where management continued by the trauma or orthopaedic surgeon.

*Inclusion criteria* were as follows: all patients above 18 years of age who presented to the emergency department with an ankle fracture. All types of ankle fractures, with the exception of avulsion fractures, were included. Both fractures needing reduction and fractures initially deemed stable were included. This allowed for an evaluation of the quality of reduction as well as an assessment whether fractures that were deemed stable remained stable following splinting.

All types meaning; Weber A or Lauge-Hansen Supination-Adduction (SA; except for small fragments meeting the exclusion criteria because they were considered ligament lesion or ankle sprain); Weber B or Lauge-Hansen Supination-Exorotation; and Weber C/Lauge-Hansen Pronation-Exorotation (except for PER 1, which meets the exclusion criteria).

*Exclusion criteria* were as follows: all patients with (solitary) avulsion fragments less than 10 mm; patients below 18 years of age; contusion or distortion ankle/no fracture; tibial fractures above the ankle joint; fractures of both tibia and fibula above the ankle joint; solitary fractures of the medial malleolus; osteomyelitis; a (concomitant) foot fracture, first radiograph from abroad, bad quality, no first conventional radiograph without splint, or first radiograph was computed tomography (CT)-scan; and when there was no follow-up at all (eg, only 1 conventional radiograph made, CT or conventional radiograph at operation room as second radiograph/directly to operation room).

### Ethics

Prior to this study approval by the institutional review board was obtained. This study was not subject to the Medical Research Involving Human Subjects Act (WMO) as it was a retrospective study (waiver No. W16-162 No. 16.189).

### Outcomes

The primary outcome was the frequency of secondary displacement at conventional radiographs after applying a splint in adult patients presenting to the emergency department with an ankle fracture, as determined retrospectively by an emergency physician and a foot ankle expert trauma surgeon. The emphasis of the analysis was to assess stable ankle fractures not requiring reduction and to identify how frequently secondary fracture displacement occurred after applying a splint.

Secondary outcome was to assess how successful reduction attempts were in the emergency department.

### Baseline and Injury Data

Baseline criteria that were recorded were as follows: patient characteristics, including age, sex, affected side of injury, timing of post-splinting conventional radiographs (in the emergency department or in the outpatient trauma clinic), and fracture characteristics, including classification according to the Lauge-Hansen and the Weber classification systems (see Table 1). Finally, it was noted whether closed reduction in the emergency department or surgery was performed and whether or not this reduction was successful. Fractures that did not need a reduction were assessed for maintaining their alignment following splinting.

When a splint was applied, the sequence overall was to first apply a stockinet, then sufficient padding, and a backslap of 12 to 15 layers of plaster of paris. The splint was molded with the palm of the hand, while the ankle was held in 90° dorsiflexion and the knee in 90° flexion over the edge of the table. Finally, when the molding was finished, the splint was secured with a bandage.

### Data Collection and Radiographic Measurements

All data were recorded utilizing the electronic emergency department patient charts or from the outpatients’ clinic follow-up and were documented in standard case report forms. Patients were always seen at the emergency department and consecutively at the outpatient clinic for follow-up. Cases were selected out of a preexisting database of patients who had an International Classification of Diseases, 10th Revision (ICD-10) coding of an ankle fracture, which was part of a previous study from one of the authors considering wound complications. The data presented in this paper had no overlap with the previous study. Moreover, to this day, there have been no significant changes in the quality of imaging, data, or practice patterns.

**Figure 1.**
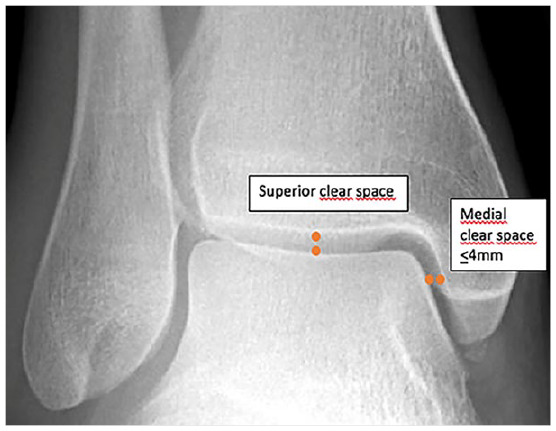
Ankle radiograph demonstrating measurements of medial clear space and superior clear space.

Regarding the radiographic measurements, mortise anteroposterior and lateral ankle radiographs had been obtained in all patients, as a standard of care. All radiographs were retrospectively evaluated on a high-resolution diagnostic monitor and a virtual ruler was utilized in a magnified digital image. All measurements were recorded in millimeters (mm) and completed to the nearest mm. Measurements on anteroposterior views included the MCS, the amount of fibular dislocation/lateral diastasis and the distal fracture height. Lateral radiographs were mainly used for observing a tertius fragment (ie, posterior malleolus fragment). The MCS was measured as the distance from the medial border of the talus to the lateral border of the medial malleolus on a line parallel to the medial articular surface, half the distance between the talar dome and the inferior aspect of the medial articular surface (Figure 1). All images were interpreted and all measurements performed by Two investigators (LW: emergency physician and TS: trauma surgeon) independent of each other and both were blinded for patient demographics.

A stable ankle fracture was defined as a distal fibular fracture without evident medial instability (no signs of rupture/injury of the medial deltoid ligament or mortise incongruency, including pain on palpation of medial malleolus) and without talar shift or tilt (measured as an increased MCS more than 4 mm or a MCS minus superior clear space of 2 mm or more).

Acceptable alignment was defined as alignment at the initial radiograph that would either refer to a stable fracture, without the need for reduction or would not affect the surgical outcomes negatively and therefore there was no need for another attempt of applying the splint at the emergency department.

### Statistical Analysis and Sample Size Calculation

In cited literature, there is only one article that shows 2% of routine radiographs leading to a change in therapy. Other studies do not show specific numbers about the percentage displacement. A displacement of 1% to 5% was accepted with a power of 80% and a 2-sided significance level of .05. With that, the total population should consist of at least 150 patients. As the percentage of displacement and therefore the power was estimated, all patients with an ICD-10 coding of an ankle fracture were selected and cases in a study period of 4 years were recruited to ensure there were sufficient numbers.

All data were presented as absolute numbers, including percentages or proportions, means with standard deviations and 95% confidence intervals (CIs), where appropriate. Numerical data without normal distribution were presented as medians and percentiles. Normality of continuous data was assessed by inspecting the distribution histograms as well as performing the Shapiro-Wilk test of normality. Differences in ankle fracture characteristics between the first and post-splinting conventional radiographs were analyzed with the McNemar test, utilizing cross tabulation. Statistical significance was defined as a *P* value of less than .05. All data were analyzed using SPSS Version 25 (SPSS, Inc., Chicago, Ill).

## Results

### Baseline Characteristics

**Figure 2.**
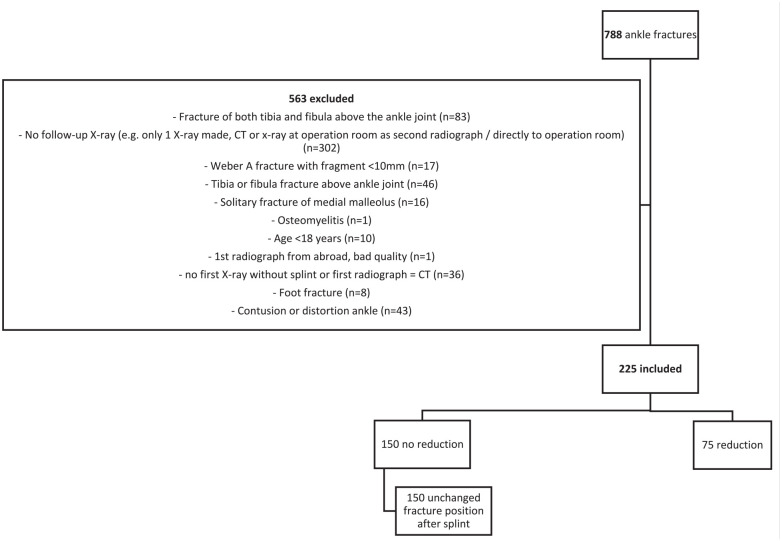
Patients flow chart. Abbreviation: CT, computed tomography.

A total of 788 adult patients presented to the emergency department with an ankle fracture during the selected study period based on the ICD-10 coding, of whom 563 were excluded as they met the aforementioned exclusion criteria (see Figure 2). This resulted in a study population of 225 patients.

Baseline characteristics of these patients are shown in Table 2. The majority of patients were female, with an ankle fracture on the right side. Most ankle fractures were classified as a Supination-External Rotation (SER) type 2, according to the Lauge-Hansen classification. According to the Weber classification, most fractures were Weber B fractures.

**Table 2.** Baseline Characteristics.

Demographic characteristics	Total (n = 225)
Female sex—n (%)	131 (58.2)
Median age—years (Q1-Q3)	52.0 (34.5-64.0)
Affected side of injury—n (%)	
Left	105 (46.7)
Right	120 (53.3)
Fracture classification—n (%)	
Lauge-Hansen—n (%)	
Supination-adduction (SA)	5 (2.2)
SA-1	4 (80.0)
SA-2	1 (20.0)
Supination-external rotation (SER)	197 (87.6)
SER-2 SER-3	116 (58.9) 12 (6.1)
SER-4	69 (35.0)
Pronation-external rotation (PER)	23 (10.2)
PER-2	1 (4.3)
PER-3	10 (43.5)
PER-4	12 (52.2)
Weber—n (%)	
A	5 (2.2)
B	197 (87.6)
C	23 (10.2)
Fracture reduction in ED—n (%)	
Yes	75 (33.3)
No	150 (66.7)
ORIF—n (%)^a^	
Yes	97 (43.1)
No	125 (55.6)

Baseline characteristics of all included patients.

Abbreviations: ED, emergency department; ORIF, open reduction and internal fixation; Q1 and Q3, first and third quartile.

a Data regarding surgical treatment were missing in 3 patients.

Of all included patients, 55 patients had radiographic signs of medial instability, resulting in 170 patients with a stable ankle fracture according to the definition mentioned above (Table 3). Moreover, 40.4% (91/225) of recruited patients showed signs of fibular dislocation of more than 2 mm and 31.1% (70/225) had a posterior ankle fracture.

**Table 3.** Radiographic Characteristics, Comparison Between Initial and Post-Splinting Conventional Radiographs.

	Fracture reduced (n = 75)			Fracture not reduced (n = 150)		
Parameter	Initial X-ray n (%)	Post-casting X-ray n (%)	*P* value	Initial X-ray n (%)	Post-splinting X-ray n (%)	*P* value
Fracture position “acceptable”			.003			.45
Yes	30 (40.0)	45 (60.0)		139 (92.7)	142 (94.7)	
No	45 (60.0)	30 (40.0)		11 (7.3)	8 (5.3)	
Medial clear space widened			.001			.38
Yes	46 (61.3)	33 (44.0)		9 (6.0)	6 (4.0)	
No	29 (38.7)	42 (56.0)		141 (94.0)	144 (96.0)	
Fibular dislocation/lateral diastasis			.18			.69
>2 mm	3 (70.7)	48 (64.0)		38 (25.3)	36 (24.0)	
≤2 mm	22 (29.3)	27 (36.0)		112 (74.7)	114 (76.0)	

### Outcomes

Post-splinting conventional radiographs were performed in all the included patients at any time point during clinical follow-up. In 78.7% (177/225) of all the included patients, the first post-splinting conventional radiograph was performed during the initial presentation, while still in the emergency department immediately after applying the plaster splint. In the other patients, the first post-splinting conventional radiograph was performed in the outpatient clinic as follows: within 3 days in 4.4% (10/225) patients, between 3 and 10 days in 13.3% (30/225) patients, and after 10 days in the remaining 3.6% of patients (8/225).

### Fractures With Changes in Alignment Post-Splinting

In general, in 51 of 225 ankle fractures, differences in fracture characteristics were detected in post-splinting radiographs. Of these 51 patients, in 40 patients (78%) the fracture position improved and in 11 patients (22%) there was loss of alignment. Ten of the latter were classified as unstable Weber B fractures before splinting and underwent reduction and surgery subsequently. The other patient had a stable ankle fracture and the position of the fracture had improved during the radiological evaluation in the outpatient clinic. Conservative fracture treatment was initiated utilizing a plaster splint. Nine radiographs out of 40 that showed difference in alignment improved without the need of reduction of which 6 were classified as stable fractures and thus with a normal MCS. The improvement was only minor; therefore, it was not pertinent for patient’s management moving forward. From this perspective, it cannot be stated whether it is of any clinical relevance. The remaining 31 radiographs showed improvement in all who underwent reduction.

### Fractures Requiring Reduction

Of all the included patients, a total of 75 patients underwent fracture reduction while in the emergency department. This was performed in 84% (46/55) of patients who showed signs of medial instability (and therefore unstable fractures) and in 17.1% (29/170) of patients with stable fractures (normal MCS). However, these 29 cases of stable fractures consisted of 15 Lauge-Hansen SER 4/Weber B fractures and, consequently, all had an operation indication. Furthermore, 13 out of the 29 comprised 12 Lauge-Hansen SER 2/Weber B fractures and 1 Lauge-Hansen PER 3/Weber C. Of the 13 SER 2 fractures, only 3 had no a fibular dislocation of more than 2 mm, and it was unclear why they had been reduced. Three of the 12 fractures were reduced either because of comborbidities (ie, diabetes and charcot) or there was doubt about the fibular dislocation. The remaining fractures were later described as SER 4 or were operated because of substantial fibular dislocation. Of all patients who underwent fracture reduction, 79% (59/75) had surgery subsequently. Of the patients who did not require fracture reduction, 14% (21/150) were subsequently operatively treated. In 150 ankle fractures, there was no fracture manipulation when applying the splint.

Differences between the initial radiograph and the first post-splinting radiograph are shown in Table 3, taking fracture reduction into account. When comparing the initial radiograph with the post-casting radiograph in patients in whom fracture reduction was required, frequencies of widening of the MCS decreased from 61% (46/75) to 44% (33/75) and the frequency of fibular dislocation decreased from 71% (53/75) to 64% (48/75).

### Fractures Not Requiring Reduction

Regarding the 150 patients who did not require fracture reduction, there were no significant differences (or improvement in fracture position) between the initial and the post-splinting ankle radiograph (*P* = .38 for MCS widening and *P* = .69 for fibular dislocation). Table 4 shows the characteristics of these patients. This group mainly consisted of patients with SER type 2 fractures (n = 102/150 or 68%) according to the Lauge-Hansen classification or Weber B (n = 134/150 or 89.3%) ankle fractures.

**Table 4.** Characteristics of Improved or Unchanged Fractures Not Requiring Reduction.

Demographic characteristics	Total (n = 150)
Female sex—n (%)	89 (59.3)
Median age—years (Q1-Q3)	54.0 (33.0-65.0)
Fracture classification—n (%)	
Lauge-Hansen—n (%)	
Supination-adduction (SA)	5 (3.3)
SA-1	4 (80.0)
SA-2	1 (20.0)
Supination-external rotation (SER)	134 (89.3)
SER-2	102 (76.1)
SER-3	11 (8.2)
SER-4	21 (15.7)
Pronation-external rotation (PER)	11 (7.3)
PER-2	1 (9.1)
PER-3	5 (45.5)
PER-4	5 (45.5)
Weber—n (%)	
A	5 (3.3)
B	134 (89.3)
C	11 (7.3)

Table showing characteristics of patients in whom the ankle fracture was not reduced. Moreover, the post-splinting X-ray showed improved or unchanged fracture positions.

Abbreviation: Q1 and Q3, first and third quartile.

In the group of patients in whom the ankle fracture was reduced, the post-casting radiograph showed a different position in 39 patients (39/75 = 52%). In 80% (60/75) of these patients, fracture positions improved and in 20% there was loss of alignment (15/75).

All fractures classified as SA type 1 (4 patients) and type 2 (1 patient) did not require reduction and remained unchanged in the post-splinting radiograph. Regarding all SER type 2 (Weber B) ankle fractures (116 patients), 14 aforementioned patients (14/116 = 12.1%) underwent reduction and 102 (102/116 = 87.9%) did not. In SER types 3 and 4, 1 patient (1/12 = 8%) and 48 patients (48/69 = 70%) required reduction, respectively. In the patients’ group with PER type fractures, there was 1 patient with type 1 who did not undergo reduction, of 10 patients with type 3 50% (5/10) underwent reduction, and of the 12 patients with PER type 4, 58% (7/12) underwent reduction.

## Limitations

A substantial limitation in this study was the difficulty of defining “stable” fractures and “acceptable alignment.” The definition of “stable fractures” was based on current literature and there is a fairly comprehensive consensus opinion that MCS widening or medial deltoid ligament incompetence is the most important indication of an unstable fracture. However, there was discrepancy between clinical management and results of this study in defining stability in a subset of patients. On the contrary, it is unclear whether this is of any clinical relevance.

In addition, it was difficult to define “acceptable alignment,” which probably created a certain amount of interobserver bias. For example, it could either mean that the fracture would be in need of surgery or that the alignment was acceptable to wait for surgery after splint application. It was supposed that alignment at the initial radiograph would not affect the surgical outcomes negatively. On the contrary, there were a few ankle fractures, without an indication to perform surgery, which was defined as acceptable alignment at the post-splinting radiograph. Although the alignment was not ideal, additional splinting or reduction attempts would not change or improve outcome.

Furthermore, in our clinical practice, nonstress views of the ankle were utilized. This could be of influence when using the Weber classification because the stability of Weber B fractures is predicated on stress views. These views might reveal widening of the mortise and thus instability. For this matter, we mainly focused on the Lauge-Hansen classification system.

Finally, this was a retrospective, single-center study with its inherent bias due to documentation bias and potential confounding factors. For example, it could be possible that reduction of the fracture had taken place but was not recorded in the electronic patient chart.

As follow-up was limited, the long-term results cannot be presented.

## Discussion

The aim of this study was to evaluate how frequently secondary displacement occurred at conventional radiographs after applying a splint in adult patients presenting to the emergency department with an ankle fracture, so as to identify the specific fracture characteristics and to assess reduction attempts in the emergency department.

The study results show that in patients with ankle fractures who did not require fracture reduction, the position of the fracture was the same or actually improved in all cases. Therefore, the need and significance of post-splinting conventional radiographs in these patients is questionable. The majority of these patients comprised those with Lauge-Hansen SER type II ankle fractures or Weber B ankle fractures. The most important feature of these fractures was the absence of widened MCS, indicating fracture stability.

Post-splinting imaging of nondisplaced fractures remains a controversial topic in health care.

One of the key reasons to obtain these radiographs in teaching institutions is to judge the position of the ankle, either after reduction to assess the quality of the reduction or after initially deemed stable fractures, to assure maintaining the correct position after manipulation of applying the cast.

There have only been a few studies focusing on the necessity of performing post-splinting radiographs. One recent retrospective observational study, also conducted in the Netherlands, evaluated the effect of routine radiographs in ankle fractures.^23^ They concluded that routine radiographs seldom change treatment strategies. Nevertheless, this study examined radiographs taken during the follow-up period, rather than in the emergency department. In 2004, Martin^19^ examined 53 patients with a Weber B fracture, without demonstrable medial instability or mortise incongruity and therefore did not require closed reduction or operative treatment. The authors concluded that only 1 initial radiograph was required, instead of a total of 6 conventional radiographs, which had been obtained previously. However, post-casting conventional radiographs were performed during follow-up at the outpatient clinic.

The authors of another study, published in 2009, showed that none of the 121 patients with nondisplaced ankle fractures were displaced on follow-up radiographs obtained at the outpatient clinic.^2^

This study is the first and largest study to suggest that post-splinting conventional radiographs for stable ankle fractures without widened MCS are unnecessary in the emergency department. In our study population, no change in immediate emergency department management was required after repeated radiographs. Our findings are supported by the results of previous studies.^4,20-24^ However, it must be noted that these studies comprised only small numbers of patients. The largest study, conducted by Chaudry et al^21^ showed that among 204 nondisplaced fractures, none had differences in fracture alignment after splinting. However, only 44 ankle fractures were included in this study because other fracture types were evaluated as well.

In summary, the results of this study suggest that post-splinting conventional radiographs in SER type 2 (without widened MCS or need for reduction) and SA type 1 and 2 ankle fractures in adult patients presenting to the emergency department are unnecessary. Moreover, omitting these conventional radiographs from routine clinical daily practice potentially decreases emergency department health care costs, and radiation exposure in individual patients can be minimized, although clinical benefit would be potentially minimal at best.^23,24^ Nevertheless, it is clinically relevant that, in these patients, emergency department length of stay potentially decreases because waiting times for radiology could add up to total length of stay. As crowding is an important and relevant issue nowadays, the study results might help decreasing this to some extent. Future research on this topic could help to improve these issues.
